# PDADMAC/PSS Oligoelectrolyte Multilayers: Internal Structure and Hydration Properties at Early Growth Stages from Atomistic Simulations

**DOI:** 10.3390/molecules25081848

**Published:** 2020-04-17

**Authors:** Pedro A. Sánchez, Martin Vögele, Jens Smiatek, Baofu Qiao, Marcello Sega, Christian Holm

**Affiliations:** 1 Laboratory of Mathematical Modeling of Physical and Chemical Processes in Multiphase Media, Institute of Natural Sciences and Mathematics, Ural Federal University, 620000 Ekaterinburg, Russia; 2 Wolfgang Pauli Institute c/o University of Vienna, 1090 Vienna, Austria; 3 Department of Computer Science, Stanford University, Stanford, CA 94305, USA; mvoegele@stanford.edu; 4 Institut für Computerphysik, Universität Stuttgart, 70569 Stuttgart, Germany; smiatek@icp.uni-stuttgart.de (J.S.); holm@icp.uni-stuttgart.de (C.H.); 5 Chemical Sciences and Engineering Division, Argonne National Laboratory, Argonne, IL 60439, USA; qiaobf@gmail.com; 6 Forschungszentrum Jülich GmbH, Helmholtz Institute Erlangen-Nürnberg for Renewable Energy (IEK-11), Fürther Str. 248, D-90429 Nuremberg, Germany; m.sega@fz-juelich.de

**Keywords:** polyelectrolyte multilayers, layer-by-layer deposition, hydration properties, charge compensation, molecular dynamics, atomistic simulations

## Abstract

We analyze the internal structure and hydration properties of poly(diallyl dimethyl ammonium chloride)/poly(styrene sulfonate sodium salt) oligoelectrolyte multilayers at early stages of their layer-by-layer growth process. Our study is based on large-scale molecular dynamics simulations with atomistic resolution that we presented recently [Sánchez et al., *Soft Matter* **2019**, 15, 9437], in which we produced the first four deposition cycles of a multilayer obtained by alternate exposure of a flat silica substrate to aqueous electrolyte solutions of such polymers at 0.1M of NaCl. In contrast to any previous work, here we perform a local structural analysis that allows us to determine the dependence of the multilayer properties on the distance to the substrate. We prove that the large accumulation of water and ions next to the substrate observed in previous overall measurements actually decreases the degree of intrinsic charge compensation, but this remains as the main mechanism within the interface region. We show that the range of influence of the substrate reaches approximately 3 nm, whereas the structure of the outer region is rather independent from the position. This detailed characterization is essential for the development of accurate mesoscale models able to reach length and time scales of technological interest.

## 1. Introduction

Layer-by-layer (LbL) deposition is one of the most relevant methods currently available for the synthesis of soft materials from ionic building blocks [1,2,3]. This method has attracted a great attention in recent decades due to its practical simplicity: when a charged substrate is exposed to a dispersion of oppositely charged molecular components, being that nanoparticles or polyelectrolytes, the latter are electrostatically adsorbed on the substrate, forming a thin layer. Under proper conditions, the surface charge of the deposited layer overcomes that one of the underlying substrates, an effect known as charge overcompensation. When the latter happens, the film can be exposed to another dispersion of oppositely charged polymers or particles, resulting in the adsorption of a second, oppositely charged layer. By iterating this alternating deposition of anionic and cationic substances it is possible to grow complex composite coatings with prescribed layered nanostructure and thickness. The properties of these multilayers, determined by the choice of the deposited materials and growth conditions, are extremely diverse. This opens up numerous possibilities for their use in multiple applications [4,5,6,7].

The usage of polyanions and polycations as building blocks in LbL processes is a choice largely explored experimentally in recent years. The resulting coatings, known as polyelectrolyte multilayers (PEMs) [8,9,10], have shown a great potential for many application fields, including the creation of biosensors and non-linear optical materials [11,12,13], catalysis and filtration membranes [14,15,16,17,18], protective and biocompatible coatings [19,20], or nanocapsules and drug delivery systems [21,22], to mention some examples. This considerable growth of experimental studies, stimulated by the generality and relative simplicity of the LbL procedure, has been accompanied by large research efforts on the fundamental understanding of PEMs assembly mechanisms. Unfortunately, not all relevant details of the internal nanostructure of the multilayers are reachable by direct observation methods [23,24]. The complexation of the polyelectrolytes during the LbL process can lead to strong structural correlations and significant intermixing between different layers. This represents a serious challenge for analytical models, which usually are forced to rely on strong approximations and assumptions on the nature of the internal structure [25]. The complexity of the interactions and the broad range of lengths and time scales involved in the behavior of these systems makes also rather difficult their computer simulation modeling. Until very recent years, most computational studies have been based on top-down coarse-grained simulation models with very crude approximations [26,27]. This strategy allows the reaching of mesoscopic time and length scales, facilitating the direct comparison with experimental measurements [26,28,29,30,31,32,33]. However, in general such models do not ensure a proper connection of the experimental observables to the underlying microscopic mechanisms governing these systems, as the latter are often simplified by means of not fully grounded assumptions. As an important example of this type of issues, it has been assumed for a long time that electrostatics is the main factor determining the complexation of polyelectrolite pairs during PEMs growth. Therefore, most simple simulation models have focused on electrostatic interactions solely, taking the solvent as a continuous implicit background with given permittivity. However, experimental findings showed that entropic ion pairing effects are fundamental in such complexation process, as the association energy is determined by the expelling of counterions and water molecules from the hydration shell when polyanion-polycation pairs are formed [34,35]. On the other hand, microscopic mechanisms can be, in principle, captured by atomistic computer simulations. Their much larger computational cost strongly limits the scales they can reach, making the comparison with experiments more difficult [26]. In addition, the LbL method involves strictly sequential steps that systematically increase the total amount of material in the system. For these reasons, most related atomistic simulations to date have been devoted not to the LbL formation of PEMs but to the study of the single step complexation of fixed small quantities of polyelectrolyte chains, usually far from any interface [36,37], or to the single step adsorption of one layer or polyions on a charged substrate [27,38].

Several years ago, we introduced the first multistep atomistic simulation protocol for the study of the LbL growth of a PEM system [39]. This protocol, briefly described in Section 3, mimics every step of the experimental procedure. In order to decrease the computing cost to a level that makes the analysis of some mesoscopic properties feasible, we focused on PEMs formed by low molecular weight polyions, or oligoelectrolyte multilayers (OEMs). Besides the computational advantage of the reduced number of atoms involved in OEMs, such systems have an intrinsic interest on their own, as their properties are expected to be different from their high molecular weight polyelectrolyte counterparts. Thus, the degree of polymerization of the building blocks can be used as an additional control parameter of the final properties of the films. With this approach we first studied the formation of bilayers of one of the most broadly used polyanion/polycation pair: poly(diallyl dimethyl ammonium chloride) (PDADMAC) and poly(styrene sulfonate sodium salt) (PSS), adsorbed on a flat substrate [39,40]. The simulations showed that the structure of such small multilayer is still dominated by the closeness of the substrate. More recently, we extended our atomsitic simulations to obtain a system of four layers of the same oligoelectrolytes, (PDADMAC/PSS)_2_, with a degree of polymerization DP=30, deposited from water solutions with 0.1M of NaCl on a charged silica substrate and subsequently rinsed with pure water. The size of this system was large enough to obtain some mesoscopic properties—as the surface roughness, film thickness and quantity of adsorbed material—comparable to experimental measurements. A good agreement between experiments and simulations was observed [41]. Stimulated by this agreement, very recently we presented a first analysis of several microscopic overall properties of such system [42] that in most cases remain inaccessible to experimental observations. Oligoelectrolyte density profiles demonstrated the development of three structural regions, as predicted qualitatively in different previous works [3,43,44]: a strongly layered region next to the substrate, a dense central region and an outermost region of smoothly decaying density. Except in the first region, the internal structure of the multilayer turned out to be rather fuzzy, with a strong complexation between the different deposited layers. An overall analysis of the charge pairing revealed that the main charge compensation mechanism in this system is intrinsic, i.e., by polyanion/polycation pairing. Differences in the structure of individual oligoelectrolyte chains with respect to infinite dilution conditions and the effect of different ionic concentrations in the rinsing solvent were also discussed.

Besides providing information on different microscopic properties [42], our most recent study on the four layers system raised several important questions that could not be addressed by means of the conventional overall analysis of the simulation data that we employed there. Such questions, which are the main subject of this work, are the following. First, the accumulation of water and ions next to the substrate revealed by the density profiles [42] suggests that intrinsic charge compensation could be much weaker in such region, but a direct proof of the existence and extent of such effect requires a local correlation analysis. In the same way one can determine whether the smoother but also significant increase of water and ions in the outermost region of the multilayer that was also observed has a similar impact. In addition, a comprehensive and detailed correlation analysis of charge pairings requires explicit consideration of the hydration of the polyion charged groups, a point that was not addressed so far. Finally, the characterization of the local properties at different positions within the multilayer will allow us to determine the range of influence of the substrate. The latter is essential to choose the atomistic information that may serve to the future development of accurate mesoscale models, based on bottom-up approaches, intended to overcome the limitations in length and time scales of full atomistic simulations.

To perform a meaningful local correlation analysis that addresses the questions described above we must take into account the symmetry of our system. Relevant variations in the properties should manifest only along the growth axis of the multilayer. Therefore, here we analyze how the hydration properties of the oligomers and the different charge pairings in the system, as the main atomistic property determining the film structure, depend on the distance to the substrate. These results are obtained for three different concentrations of NaCl dipping solutions, c={0,0.1 M,0.5 M}, to which the films are exposed after the four deposition cycles are performed. Results for these systems are also compared to that of single oligomer chains in bulk solutions of the same ionic strength.

## 2. Results and Discussion

**Figure 1:**
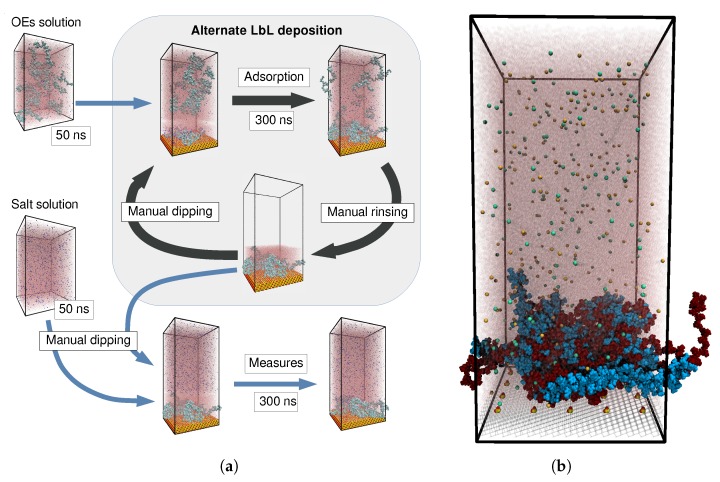
(**a**) Scheme of the simulation protocol used to reproduce the main steps of the LbL growth process. (**b**) Simulation snapshot of an OEM system obtained after four deposition cycles and exposure to a solution of 0.1 M of NaCl. PDADMAC oligomers are colored in dark red, PSS ones in light blue. Na^+^ and Cl^−^ ions are colored in pale orange and blue, respectively. The silica substrate is represented as a wireframe, except for its surface charged groups. Water molecules are translucent to ease the visualization.

Figure 1a shows a scheme of the main steps of our simulation protocol for the build-up of an OEM. These steps are intended to mimic the experimental LbL growth procedure. As an example of their outcome, Figure 1b shows a typical system snapshot obtained after four deposition cycles and subsequent exposure of the resulting film to a solution of 0.1 M of NaCl. Since the silica substrate has a negative surface charge, the first and third deposition cycles correspond to PDADMAC chains (depicted in dark red color), whereas second and fourth correspond to PSS ones (in light blue). The fuzzy structure of the film, with no clear layering derived from the alternating deposition of each type of oligomers, can be observed. Detailed explanations of this simulation protocol and of the one used for systems of single chains are included in Section 3.

We start the detailed analysis of the properties of the (PDADMAC/PSS)_2_ system by calculating the radial distribution functions (RDFs) corresponding to the most important group pairings. In order to determine how such pairings change along the growth axis, in each calculation the positions of the reference groups are partitioned into thin overlapping slices parallel to the substrate surface. A thickness of Δh=1 nm is taken for these slices, which are placed at distances to the substrate ranging from h=0.3 nm to h=5.8 nm, with intervals of 0.5 nm. The upper limit corresponds approximately to the external border of the central region of the film [42]. The RDF of atoms of group β around a reference group α located inside the slice *h* is defined as

(1) $$g\left(r;\alpha /\beta ,h\right)=\langle \frac{\sum_{ij}\delta (∥{\vec{r}}_{i}-{\vec{r}}_{j}∥-r)}{N_{\alpha }\left(h,\Delta h\right)4\pi r^{2}\rho_{\beta }}\rangle ,$$

where δ is the Kronecker delta function, the index *i* runs over each atom from group α whose distance to the substrate lies between h−Δh/2 and h+Δh/2, $N_{\alpha }\left(h,\Delta h\right)$ is the number of those atoms, index *j* runs over all atoms of type β and $\rho_{\beta }$ is the overall number density of the latter, which is calculated as the integral along the *z* direction of its density profile within the region occupied by the multilayer. The statistical average 〈·〉 is performed over all measurements (see Section 3 for the corresponding details). In order to apply the definition (1) to our finite simulation dataset, we performed a binning of distances $∥{\vec{r}}_{i}-{\vec{r}}_{j}∥$, so that

(2) $$g\left(r;\alpha /\beta ,h\right)=\langle \frac{\sum_{ij}\Theta_{\Delta r}(∥{\vec{r}}_{i}-{\vec{r}}_{j}∥-r)}{N_{\alpha }\left(h,\Delta h\right)\frac{4}{3}\pi \left[{\left(r+\Delta r/2\right)}^{3}-{\left(r-\Delta r/2\right)}^{3}\right]\rho_{\beta }}\rangle ,$$

where $\Theta_{\Delta r}\left(r\right)$ is a binning function whose value is 1 when |r|<Δr/2 and 0 otherwise. Please note that Equation (2) tends to (1) in the limit of zero bin width, Δr→0.

**Figure 2:**
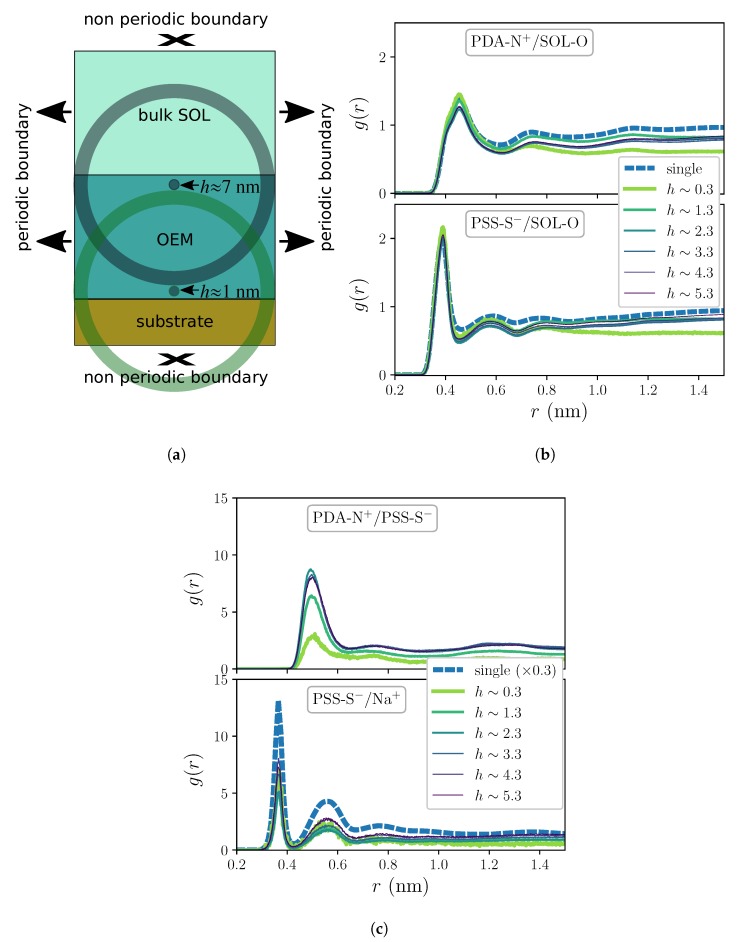
(**a**) Sketch of the system illustrating the maximum range at which one can sample correlation functions and their different bias depending on the distance to the substrate of the reference position, *h*. (**b**,**c**) Selected RDFs of different pairs obtained for single chains and for the OEM system when exposed to pure solvent conditions. Curves for OEM correspond to different distances to the substrate, *h*, calculated from a partitioning of the positions of the reference groups in horizontal slices of thickness Δ*h* = 1 nm. (**b**) Distributions of waters around PDADMAC (upper panel) and PSS (lower panel) charged groups. (**c**) Distributions obtained for each charge pairing within the film.

Besides the horizontal slicing for the sampling of the reference positions, expressions (1) and (2) correspond to standard definitions of pair correlation functions. In general, the normalization of these functions makes them to asymptotically converge to 1 when the system is isotropic far from the region of interest. However, the particular anisotropy and limited size of our system makes the asymptotic behavior unreachable for any meaningful range of distances one can sample. This is illustrated by the sketch presented in Figure 2a, in which we show the three main regions of the simulation box: from top to bottom, the “bulk” solvent region (bulk SOL), the polymer multilayer (OEM) and the solid substrate. Please note that the width and height of the sketched OEM and substrate regions keep approximately the same proportion as in the actual simulation box. The two semitransparent circular regions, centered respectively at reference points located next to the substrate surface and to the free surface of the multilayer, represent the maximum radius of the spherical shell-shaped bins one can use to calculate the correlation functions without using lateral periodic image positions of the atoms, which is statistically meaningless. These circular regions evidence that the content of the bins depends strongly on the reference position: while sampling correlations close to the free surface of the multilayer includes a significant volume fraction of the bulk solute region, the sampling close to the substrate is biased by a large fraction with no solvent nor polymers, as large bins overlap with the solid substrate and can even go beyond the border of the simulation box in the direction in which its size is strictly finite (that is, no periodic boundary is applied). However, it is important to underline that this effect on the behavior of the correlation functions at distances that compare to the lateral size of the simulation box is irrelevant for our discussion, as here we are interested only on the first coordination shells, whose maximum range is in all cases shorter than ∼0.7 nm.

**Figure 3:**
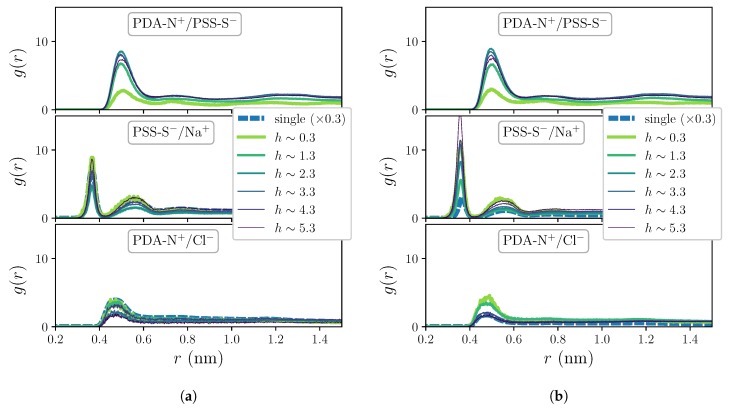
Selected RDFs for polyions charge pairings corresponding to systems under non-zero added salt concentration conditions: *c* = 0.1 M (**a**) and *c* = 0.5 M (**b**).

Figure 2b,c show the RDFs obtained for systems under no added salt concentration conditions, *c* = 0, corresponding to various selected distances to the substrate, *h*. They also include the distributions obtained for the respective single chain systems. Figure 2b corresponds to the distribution of water molecules around the charged groups of the polyions within the film, -N^+^ for PDADMAC (denoted as PDA-N^+^) and -${\mathrm{SO}}_{3}^{-}$ for PSS (denoted as PSS-S^−^). Water molecules are represented by their oxygen atoms (indicated as SOL-O). Important general differences between the profiles of each pair can be clearly observed: whereas the first coordination shells of PSS-S^−^/SOL-O distributions show a relatively narrow peak at a distance slightly below 0.4 nm, the corresponding to PDA-N^+^/SOL-O is broader and shifted above 0.45 nm. The same differential trend affects next coordination shells. Compared to single chain distributions, the first coordination shells show in both cases a good quantitative agreement, whereas peaks of the next shells are slightly higher for single chains. Interestingly, one can also observe a quantitative impact of the distance to the substrate on these profiles, with small differences in the heights of the peaks depending on *h*. This effect becomes much more pronounced in the distributions for the polycation-polyanion pairing, PDA-N^+^/PSS-S^−^, shown in the upper panel of Figure 3b: one can clearly see that this pairing is significantly weaker at small distances from the substrate than at larger ones. In this case, the separation distance for the first shell, slightly above 0.5 nm, is larger than any other from the rest of group pairs considered here. Finally, the distributions for the extrinsic charge pairing PSS-S^−^/Na^+^, shown in the lower panel of the same figure, display in general a clear layering and a moderate dependence on *h*, whereas they are in general much smaller than the curve corresponding to the single chain. The latter reflects the significant preference for the intrinsic charge compensation mechanism of this PE pair over the extrinsic one, which is the only possible mechanism in the single chain system.

The RDFs for the hydration shells around polyions charged groups, PDA-N^+^/SOL-O and PSS-S^−^/SOL-O, do not show significant differences with respect to the ones in Figure 2b when the systems are exposed to the two sampled added salt concentration conditions, thus these curves are not displayed here. However, as Figure 3 shows, the impact of added salt on the charge pairing distributions (corresponding to c=0.1 M in Figure 3a and to c=0.5 M in Figure 3b) is small but not negligible, revealing quantitative differences at least for the curves of extrinsic pairings. In particular, in these plots one can see a non-monotonous dependence of the heights of the peaks of PSS-S^−^/Na^+^ distributions on *h* that is enhanced by the concentration of added salt. The presence of added salt also allows calculation of the distributions for the pair PDA-N^+^/Cl^−^, which show only one clear broad shell with a maximum at around 0.45 nm. Besides this, the impact of added salt is much weaker on the distributions of the multilayer system than on the ones of the single chain system that show a strong quantitative decrease with *c*. This further demonstrates that the structure of the OEM is dominated by an intrinsic charge compensation mechanism, which is an assumed condition for stable PE complexes and multilayers [45]. On the other hand, Figure 2 and Figure 3 underline the qualitative differences exhibited by the first coordination shells of waters and free ions around PDADMAC and PSS charged groups that are reflected by their distinct characteristic separations and thickness. Diverse experimental measurements have proven that polyion pairs with very similar structures and distances between their charged groups tend to form strongly intrinsically compensated complexes [35]. In LbL deposition processes this leads to very thin and flat layers, with very low hydration [46]. As a result, the growth of multilayer structures is very unstable or even impossible when the intrinsic compensation is too strong due to the lack of charge overcompensation in the outermost region of the film. On the contrary, structural dissimilarities of the polyion pair tend to weaken the intrinsically compensated complexation, leading to thicker, highly hydrated structures [35]. The addition of salt ions during the LbL growth also weakens the interactions between polyions, decreasing the degree of intrinsic compensation. In the OEM system considered here, the use of moderate added salt concentrations in the dipping solutions is essential to achieve a stable film growth [47].

**Figure 4:**
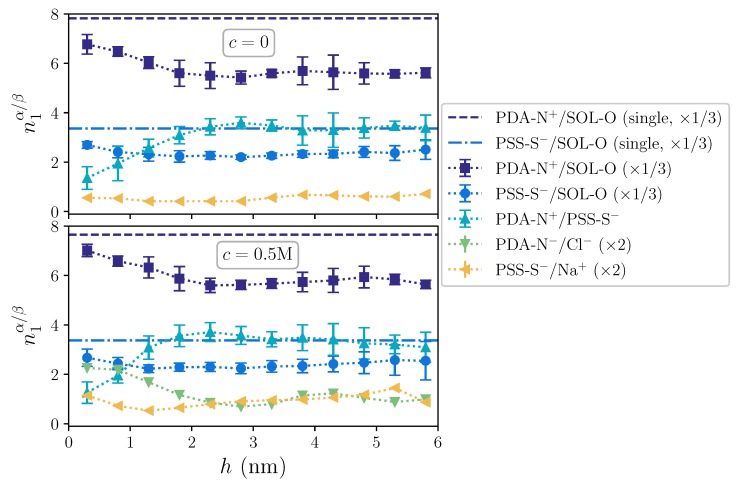
First coordination number of solvent and charges around OE charged groups as a function of the distance to the substrate, *h*, for c=0 (upper panel) and c=0.5 M (lower panel). Horizontal dashed and dashed-dotted lines indicate the hydration coordination for PDA-N^+^ and PSS-S^−^, respectively, in single chain systems. Dotted lines are a guide to the eye. Curves for solvent and ions have been rescaled to ease the comparison. Error bars of the latter are not shown for the sake of clarity.

The dependence of the pair distribution functions on the distance to the substrate demonstrates that the film has a nonhomogeneous structure along its growth axis. This can be better analyzed by calculating the coordination number of the first shell for each pair α/β, $n_{1}^{\alpha /\beta }$, as a function of *h*. This parameter is calculated by integrating the corresponding RDFs in the following way:

(3) $$n_{1}^{\alpha /\beta }=4\pi \left(\rho_{\alpha }+\rho_{\beta }\right)\int_{0}^{r_{\min}}r^{2}\;g\left(r;\alpha /\beta ,h\right)\;dr,$$

where $\rho_{\alpha }$ and $\rho_{\beta }$ are the number densities of each group obtained by averaging their number density profiles as mentioned above, and $r_{\min}$ is the position of the first minimum of g(r;α/β,h) after its first maximum. The results of these calculations are shown in Figure 4.

Even intrinsic compensation dominates along the whole film structure, as indicated by the largest coordination of PDA-N^+^/PSS-S^−^ compared to the ones of PDA-N^+^/Cl^−^ and PSS-S^+^/Na^+^, it is clearly much weaker next to the substrate, growing with *h* up to a saturation point at around h∼ 2–3 nm. With respect to this behavior, the coordination of the hydration shell for both polyions follows qualitatively an opposite profile, showing a maximum hydration next to the substrate and a decreasing one up to h∼ 2 nm. In addition, the hydration coordination of polyions in the film only approaches that of single chains, indicated by horizontal lines, in the region next to the substrate, whereas it is significantly lower in the upper region. The latter is associated with the lower hydration of intrinsically compensated polyion complexes. Finally, whereas no significant effect of the added salt concentration can be observed on the coordination of intrinsic pairing, variations in the extrinsic pairings with free ions are only significant under non-zero added salt conditions, where they exhibit a profile qualitatively similar to the one of the hydration shell. Therefore, with this analysis, we are capturing the range of influence of the substrate on the film structure: next to the substrate, on one hand, the charge compensation of polycations comes not only from free ions and complexation with polyanions, but also from the substrate surface charges; on the other hand, this surface is significantly hydrophilic and remains highly hydrated, making the water expelling associated with intrinsic polyion complexation processes more difficult. These effects smoothly vanish with the distance to the substrate surface and, approximately above ∼2 nm, the complexation is not influenced by them anymore.

Water plays a crucial role in the preferential selection of charge pairings. As a sensible coefficient for preferential interaction, we use the local/bulk partition coefficient, $K_{p}\left(r\right)$[48,49,50,51,52]. This parameter measures the preferential accumulation or exclusion of a solute around a polymer surface compared to its degree of hydration, partitioning water and solute into local and bulk regions. Specifically, we calculate this parameter for each atomic charge pairing α/β and distance to the substrate *h*, according to

(4) $$K_{p}\left(r;\alpha /\beta ,h\right)=\frac{{\langle n_{\beta }\left(r\right)\rangle }_{\alpha ,h}/{\langle n_{\mathrm{SOL}}\left(r\right)\rangle }_{\alpha ,h}}{n_{\beta }^{\mathrm{tot}}/n_{\mathrm{SOL}}^{\mathrm{tot}}},$$

where $n_{\beta }\left(r\right)$ is the number of solutes of type β at a distance not larger than *r* from α groups and $n_{\beta }^{\mathrm{tot}}$ is the total number of solutes β in the system, considering only the region occupied by the film. Analogous definitions with respect to waters apply to $n_{\mathrm{SOL}}\left(r\right)$ and $n_{\mathrm{SOL}}^{\mathrm{tot}}$, whereas ${\langle \cdot \rangle }_{\alpha ,h}$ denotes the same slicing and averaging used in Equation (2). For simplicity, here we calculate ${\langle n_{\beta }\left(r\right)\rangle }_{\alpha ,h}$ and ${\langle n_{\mathrm{SOL}}\left(r\right)\rangle }_{\alpha ,h}$ as the ratio of the corresponding mean cumulative RDFs. Please note that the local/bulk partition coefficient takes values $K_{p}\left(r\right)>1$ in regions where there is preferential accumulation of solute β around α groups, whereas $K_{p}\left(r\right)<1$ indicates preferential exclusion.

**Figure 5:**
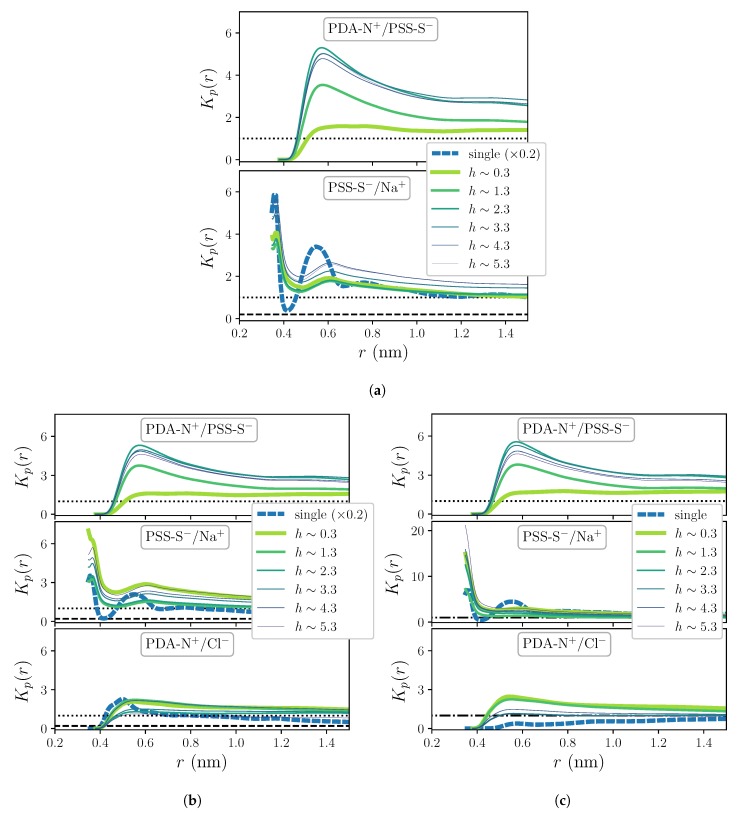
Local/bulk partition coefficient for *c* = 0 (**a**), *c* = 0.1 M (**b**) and *c* = 0.5 M (**c**). Dotted and dashed horizontal lines indicate the limit between accumulation/exclusion behavior for OEM and single chain systems, respectively, when their curves are plotted in different scales; dashed-dotted horizontal lines indicate the same limit for both systems when their curves are in the same scale.

Figure 5 shows a selection of results of $K_{p}\left(r;\alpha /\beta ,h\right)$ obtained for each considered charge pairing and added salt concentration, calculated for both OEM and single chain systems. For all concentrations, intrinsic charge pairings display a clear accumulation behavior, with a maximum at around ∼0.57 nm. Interestingly, the dependence on *h* is strongly enhanced in this parameter with respect to RDFs: for PDA-N^+^/PSS-S^−^, it shows a much weaker accumulation preference close to the substrate than far from it, whereas effects of added salt concentration are indistinguishable. However, the latter are significant for extrinsic pairings, particularly in single chain systems, which show a strong weakening of the accumulation with increasing *c*. In all cases the pair PSS-S^−^/Na^+^ presents a rather layered profile, with a first, absolute maximum at around ∼0.36 nm, which coincides with its intercharge distance for the first shell, as indicated by the RDFs (see Figure 2c and Figure 3a,b. However, the position of such maximum is difficult to determine due to the fact that the distance from PSS-S^−^ groups to their first hydration shell is slightly larger (∼0.39 nm, see Figure 2b), so $K_{p}\left(r\right)$ tends to diverge at shorter distances. Therefore, this pair will be excluded from the following discussion. Finally, the pair PDA-N^+^/Cl^−^ shows the weakest preferential accumulation behavior, being in some cases close or within the preferential exclusion regime.

**Figure 6:**
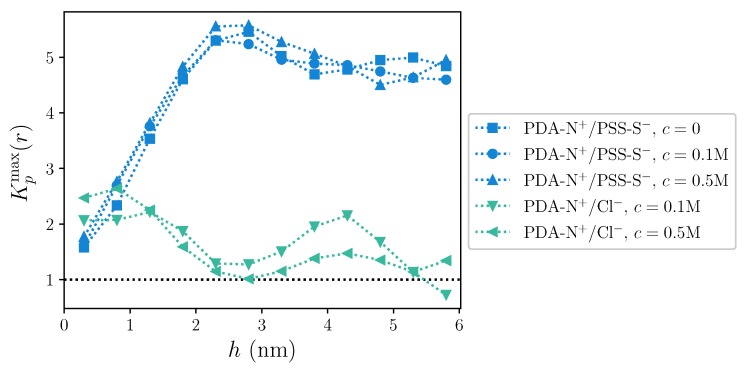
Values of the maxima of the local/bulk partition coefficient, $K_{p}^{\max}\left(r\right)$, as a function of the distance to the substrate, *h*, for PDA-N^+^/PSS-S^−^ and PDA-N^+^/Cl^−^ charge pairings. Horizontal dashed line indicates the limit between preferential accumulation/exclusion behavior. Dotted lines are a guide to the eye.

To better visualize the dependence of the accumulation/exclusion behavior for PDA-N^+^/PSS-S^−^ and PDA-N^+^/Cl^−^ charge pairings on *h*, we show in Figure 6 the values of $K_{p}\left(r\right)$ corresponding to the first maxima of the curves obtained for each sampled *h*, $K_{p}^{\max}\left(r\right)$. Here, the change with the distance to the substrate for intrinsic pairings shows very clearly the same trend that was observed for the first coordination numbers: next to the substrate, intrinsic pairing has only a slight accumulation preference; this grows significantly up to a maximum around ∼2.5 nm and slightly decays for larger distances, remaining far above the limit of preferential accumulation. This behavior seems basically independent from the concentration of added salt. The pair PDA-N^+^/Cl^−^ shows a moderate general decay of the preferential accumulation with *h*, from small values that, close to the substrate, compare to the ones of PDA-N^+^/PSS-S^−^, to the accumulation/exclusion limit at large *h*. However, this decrease is not simply monotonous, as the curves show a second maximum around h∼ 4–5 nm. Regarding the dependence on *c*, one has to take into account that in this case the denominator in (4) grows by a factor of 5 when changing from c=0.1 M to c=0.5 M. Therefore, the main changes in the accumulation/exclusion behavior with growing *c* are effectively taking place in the region close to the substrate, where the accumulation of Cl^−^ ions on polycation charged groups grows in a larger proportion than the increase of their overall density in the system.

## 3. Materials and Methods

Using molecular dynamics with atomistic resolution, we simulated the first four deposition cycles of a PDADMAC/PSS multilayer, grown on a flat silica substrate from water solutions of oligomers with degree of polymerization DP=30 and 0.1 M of monovalent added salt concentration. Experiments have shown that finite added salt concentrations are required for a stable growth with such short polyions [47]. Counterions and added salt ions in the system are Na^+^ and Cl^−^. The substrate is a flat, four layers silicate sheet with lateral periodic structure of lengths 13.1650 nm and 12.1608 nm. It is placed at z=0 in a rectangular simulation box with the same lateral lengths and periodic boundaries in the corresponding directions (*x* and *y*, see Figure 1). Its surface is made hydrophilic by replacing the uppermost oxygen atom in every silicate tetrahedron with polar hydroxil groups (−OH). These hydroxil groups are free to rotate to avoid artifacts associated with fixed orientations [39,53]. The surface possesses a negative charge distribution, obtained by skipping one in every four of the placements of the hydroxil groups, leaving exposed the ${\mathrm{SiO}}_{2}^{-}$ group. This corresponds to an average surface charge density of σ=−0.03 C/m^2^, in good agreement with the experimental values measured under pH range 6.5 to 8.5 [54,55]. Please note that the surface charge of experimental samples depends strongly on the presence of defects and pores [56]. Our choice for the placement of surface charges assumes that their local microscopic distribution has no relevance on the mesoscopic properties of the system, as has been shown in recent studies [57]. Please note that the negative charge of the substrate imposes the first and third deposition cycles to be done from PDADMAC solutions, whereas the second and fourth correspond to the PSS ones.

We calculate the interactions of the OE chains with the OPLS-AA force field [58]. Details of its parametrization for our system are found in reference [36]. For the solvent, we use the SPC/E water model due to its accuracy to reproduce the dielectric constant [59,60,61]. Since SPC/E is a rigid bond model, we apply the SETTLE algorithm [62] to constrain the covalent bonds of all water molecules. Van der Waals forces are represented by Lennard-Jones potentials shifted to smoothly decay to zero at distances larger than 0.9 nm. In order to take into account accurately the long-ranged electrostatic interactions we employ the particle-mesh-Ewald method (PME) [63,64] with a Fourier spacing of 0.125 nm and a real-space Ewald cutoff of 1.3 nm.

Our protocol includes MD simulations in the NPT and NVT ensembles. For the former, we use the semi-isotropic Parrinello–Rahman barostat [65,66], imposing a pressure of 1 bar in *z* direction. In general, temperature in the system is kept to *T* = 298 K by means of a Nosé-Hoover thermostat with a relaxation time of *τ* = 0.5 ps.

The first step of the simulation protocol (‘Manual dipping’ in Figure 1a) reproduces the dipping of the deposition wafer into one of the polyion solutions by placing a system of OEs, ions and water on top of the substrate. Such a dipping solution system (‘OE solution’ in Figure 1a), which is equilibrated in a separate simulation of 50 ns in the NPT ensemble, consists of 20 OE chains of the corresponding polyion, their respective 600 counterions (Cl^−^ for PDADMAC and Na^+^ for PSS) and 192 Na^+^ and Cl^−^ ions of added salt, solvated with 99286 water molecules. The combined system is relaxed in an NVT simulation of about 300 ns (‘Adsorption’ in Figure 1a). Being the crucial step of our protocol, such relaxation consists of several parts. The first part runs for 100 ps with an integration time step of δt=1 fs. This corresponds to around $10^{4}$ oscillations of the covalent bonds of the hydrogen atoms, thus no memory of their initial configurations should remain after this run. In the second part, the LINCS algorithm [67] is applied to constrain hydrogen covalent bonds and another run of 100 ps with time step of δt=2 fs takes place; finally, all covalent bonds become constrained with the LINCS algorithm and a production run of 300 ns with δt=2.5 fs is performed. During this process, a fraction of OE chains from the dipping solution are adsorbed on the substrate and/or on the OEs deposited in previous cycles. By taking a cutoff minimum distance of 1 nm, we checked that in all cases the number of adsorbed chains reached a saturation value within the simulation time.

The last step of each LbL deposition cycle consists of the rinsing of the film wafer with pure solvent or a pure salt solution to remove the supernatant PEs. In simulations we reproduce the rinsing with pure water (‘Manual rinsing’ in Figure 1a) by simply removing all atoms placed above the uppermost from the ones belonging to any of the adsorbed OE chains from the simulation box. In addition, we remove all added salt ions from the entire simulation box. The resulting rinsed system is used as the starting point for the next deposition cycle.

After four deposition cycles following the protocol described above, we expose the obtained multilayer to three NaCl solutions of different concentration: *c* = 0 (pure solvent), 0.1 M and 0.5 M. A final NVT run of 300 ns, with the same steps of the ‘Adsorption’ procedure, is carried out. Measures of the system properties are taken during the last 100 ns. In order to improve the statistics of such measurements, three independent runs are performed for each system.

The simulations for single chains involve a simpler protocol: first, we perform a steepest descent energy minimization for 10^3^ integration steps; second, a NVT run of 2 ns with a Berendsen thermostat and integration time step of δt=1 fs; then, a NPT equilibration step of 10 ns with the Nosé-Hoover thermostat; finally, the production run consists of an extension of the latter step to 400 ns, using an increased time step δ=2 fs.

The software packages Packmol [68], Gromacs [69] and MDAnalysis [70] were employed, respectively, for the set-up of the system, for carrying out the MD simulations and for partial analysis of the simulation data.

## 4. Conclusions

In this article, we presented the results of an extended analysis, based on local correlation measurements, of previous atomistic simulations performed to study the formation of a four layer oligoelectrolyte system created by LbL deposition on a hydrophilic substrate. This system represents an important building block to study the structure of stable polyelectrolyte multilayers.

Measurements of overall charge correlations performed in our previous work showed that on average, the main charge compensation mechanism in this system is intrinsic, whereas in density profiles we observed a significant accumulation of water and ions in a narrow region next to the substrate surface, as well as in a broader region corresponding to the free surface of the multilayer. This suggested the existence of a nonhomogeneous distribution of charge correlations along its growth axis. In order to prove and characterize such inhomogeneity, here we performed a local correlation analysis for the charge pairings and the hydration of polyion charged groups, as the main parameters determining the internal structure and stability of the multilayer. Our results confirm that the proximity of the hydrophilic substrate surface strongly weakens the intrinsic charge compensation mechanism and strengthens the polyion hydration. Intrinsic compensation (hydration) grows more quickly (decreases) with the distance to the substrate up to approximately 3 nm, which can be considered the maximum range of influence of the substrate on the film structure. At larger distances correlations remain very similar, indicating a high degree of intrinsic compensation that extends even to the region of the free surface of the multilayer. This suggests that despite the small size of our system, we might have reached a steady regime of correlations that could apply to the main regions of much larger systems. This makes these results a promising basis for the development of bottom-up designed mesoscale models that could reach the typical lengths and time scales involved in experimental systems. Finally, exposure of the multilayer to different moderate added salt concentrations does not have any qualitative impact on the observed behavior.

## Abbreviations

The following abbreviations are used in this manuscript:

LbL	layer-by-layer
PEM	polyelectrolyte multilayer
OEM	oligoelectrolyte multilayer
PDADMAC	poly(diallyl dimethyl ammonium chloride)
PSS	poly(styrene sulfonate sodium salt)
RDF	radial distribution function
